# The Level of Lymphedema Awareness among Women with Breast Cancer in the Kingdom of Saudi Arabia

**DOI:** 10.3390/ijerph18020627

**Published:** 2021-01-13

**Authors:** Fatmah Alsharif, Wedad Almutairi, Faygah Shibily, Fatmah Alhothari, Fidaa Batwa, Nidaa Batwa, Lujain Alharbi

**Affiliations:** 1 Medical Surgical Nursing Department, King Abdulaziz University, Jeddah 21589, Saudi Arabia; fshibily@kau.edu.sa (F.S.); falhothari@stu.kau.edu.sa (F.A.); fbatwa@stu.kau.edu.sa (F.B.); nbatwa@stu.kau.edu.sa (N.B.); lalharbi0050@stu.kau.edu.sa (L.A.); 2 Maternity and Pediatric Nursing Department, King Abdulaziz University, Jeddah 21589, Saudi Arabia; walmutairi@kau.edu.sa

**Keywords:** breast-cancer-related lymphedema, level of awareness, lymphedema

## Abstract

Background: Lymphedema is a condition in which excessive fluid accumulates in soft tissues. It is a common complication of breast cancer treatments. It can lead to serious consequences and interfere with the activity of daily living. This study aimed to determine the level of awareness of breast-cancer-related lymphedema (BCRL) among women with breast cancer in the Kingdom of Saudi Arabia. This was a descriptive quantitative cross-sectional design that included a convenience sample of women diagnosed with breast cancer in the Kingdom of Saudi Arabia. Data were collected by distributing a self-administrated online questionnaire consisting of four parts, including demographic data (five items), the status of education about BCRL (three items), basic medical history of breast cancer (six items), and BCRL level of awareness of risk factors and management (nine items). Results: In total, 95 out of 135 of participants did not know about lymphedema, 119 of the participants (88.1%) did not receive any explanation about the possibility of lymphedema from their medical team before surgery, and 121 of them (89.6%) did not receive it after surgery. The most significant factor affecting participants’ level of awareness regarding BCRL was the lack of information about the possibility of BCRL occurrence, which was not provided to them by the medical team. Recommendation: Early and continuous education for future management is essential to prevent problems related to BCRL and improve quality of life.

## 1. Introduction

Breast cancer is a type of cancer in which breast cells grow out of control. It is considered to be one of the most common cancers affecting women worldwide, with an incidence of 23% [1] and 28.7% in Saudi Arabia [2]. Breast cancer related lymphedema (BCRL) prevalence is stated to be 6% to 83%, meaning that multiple trials have observed dramatically different results [3]. The incidence of lymphedema in Saudi Arabia is 14.05%, as found by KAUH in Jeddah [4]. The most common complication of breast cancer treatment is BCRL, which can be described as a condition of excessive fluid accumulation in soft tissues [5] Various risk factors such as surgery, radiation treatment, chemotherapy, diagnostic period, pathological lymph node condition, infection, and weight gain are linked to the progression of BCRL [6,7,8].

Lymphedema has different causes, and it commonly occurs as a result of breast cancer treatment such as radiotherapy, chemotherapy, and lymph node dissection [5]. Lymphedema has two types: primary lymphedema has had unknown causes until now, as it has been determined that it can evolve from inadequacies in the structure or function of the lymphatic system [9]; and secondary, or acquired, lymphedema is caused by interruption compression of the lymphatic system from tumors or their treatment [9]. Lymphedema develops gradually and can be classified into stages I–III; each stage is distinguished by specific signs and symptoms [10]. Stage I is characterized by mild discomfort, a sensation of fullness in the arms, and pitting; this stage can be reversible. Stage II is characterized by nonpitting, pain sensations, thickness of skin, and hair loss; this stage is irreversible. Stage III is characterized by enlargement of affected limbs and open wounds that may progress to severe infection; it is irreversible [10].

It can be managed by multiple treatments, including compression therapy, therapeutic exercises, and pharmacotherapy [11], but the most used treatment is complete decongestive therapy [12,13,14]. Researchers have found that the level of awareness among Saudi females about breast cancer is insufficient [15]. Lymphedema awareness among women with BC could be a major factor in preventing the development of lymphedema and improving their quality of life [16].

BCRL can lead to serious consequences and have a negative impact on the quality of life of women with breast cancer in many aspects, such as psychological problems that can disturb body image, anxiety, depression, social isolation, and sexual problems [17,18,19,20,21]. Further, if BCRL is not treated or discovered early, it can cause severe physical pain for women with breast cancer that could interfere with the activity of daily living and cause limitations in their range of motion.

### 1.1. Objectives of the Study

This study aimed to determine the level of awareness of breast-cancer-related lymphedema (BCRL) among women with BC in the Kingdom of Saudi Arabia, as well as provide the basic data needed to initiate proper education.

The research question was, “What is the level of lymphedema awareness among women with breast cancer in the Kingdom of Saudi Arabia?” Therefore, our study was done to determine the level of awareness of BCRL among women with breast cancer in the Kingdom of Saudi Arabia and to determine the gap of knowledge so as to provide basic data needed to initiate an early and proper educational program about BCRL for women with breast cancer.

### 1.2. Literature Review

There are 10 studies that discuss the awareness of lymphedema among women with breast cancer. For example, one study aimed to explore the evidence for cancer-related lymphedema. The results show that physical activity and sentinel lymph node biopsy can minimize the risk of lymphedema (LE), while chemotherapy and obesity are considered as risk factors for increasing LE. Lymphoscintigraphy may increase awareness regarding LE, which can reduce the development of LE [22].

A literature review was done in the United States in 2016 to review the knowledge of public health care providers on BCRL. It was found that the long-lasting risk factors for BCRL include the following: axillary node involvement, the type of breast surgery, and radiotherapy [11]. Another study was conducted to evaluate the cognitive and emotional factors that influence adherence to the risk management of lymphedema. It was published in Philadelphia, Pennsylvania, United States. A total of 62 women at Fox Chase Cancer Center received a baseline questionnaire. After that, they received a booklet containing information about lymphedema. The questionnaires were distributed again after 6 and 12 months to reassess the two factors. The overall results indicate increased commitment, and there are clear differences between the two assessments. Knowledge increased while distress decreased, which are considered to be two major factors that increase adherence [23].

In addition, a study was conducted to explore the prevalence of lymphedema occurrence in women undergoing breast cancer treatment. It showed that the prevalence of lymphedema among 250 women undergoing breast cancer treatment was 44.8%, and this is considered to be relevant because it is a disease that affects many aspects of a patient’s overall health (psychologically, physically, and functional damage) [24]. A total of five studies were examined to explore the level of awareness of BCRL among breast cancer patients. One such study was published in Busan-Gyeongnam, Korea. In 2015, the results showed that the majority of patients with breast cancer who participated mainly fell into two categories: the first lacked awareness of BCRL, and the second had false thoughts about it, which indicates that insufficient education was provided to these patients about lymphedema [25].

Furthermore, another study was published in Business at Kaiser Permanente Northern California (2012). The results show that lymphedema awareness varied depending on the age of the breast cancer patients [16].

Likewise, a qualitative systematic review was published in Clonmel. The participants expressed their inability to engage in daily activities, thus leading to a decrease in their quality of life. The results show that the participants lacked information regarding BCRL [26]. A similar result was found in a study done in Turkey at the Norton School of Lymphatic Therapy (2016), which showed that the level of information was low, and only 35 had declared that they were aware of lymphedema; 145 patients were neither informed nor educated about BCRL. The study showed that the risk of developing lymphedema increased in patients who did not receive information about lymphedema after surgery more than in those who had received it [27]. In contrast to the above studies, a study was done in Bialystok, Poland (2014). The participants demonstrated a high level of knowledge concerning lymphedema prevention, but they did not adhere to preventive guidelines. The study showed that an assessment of quality of life should be an essential ingredient of rehabilitation for all patients, especially breast cancer, as mastectomies have an impact on the outcome during the perioperative period [28].

Further, a cross-sectional study was done in New York, United Sates, which aimed to investigate cognitive and symptomatic outcomes when providing information about BCRL. The results were as follows: 57% of participants said that they had received information on BCRL, 18% of the participants did not have symptoms of BCRL, and the participants who received information about BCRL had fewer symptoms than the participants who did not receive information regarding BCRL [29].

The researchers had to use old studies as a reference in the literature review to reach the necessary numbers of studies, and there was no adequate recent research about BCRL in general and BCRL level of awareness in particular.

## 2. Materials and Methods

### 2.1. Research Design

A descriptive quantitative cross-sectional design was used among females with breast cancer in Saudi Arabia.

### 2.2. Setting

This study was conducted in Saudi Arabia.

### 2.3. Study Sampling and Sample Size

A convenience sample of 135 voluntary participants involved in the study included adult women with breast cancer or women who were breast cancer survivors. Their ages were 18 years and above, and they lived in the Kingdom of Saudi Arabia. We excluded males who have breast cancer, women with breast cancer under 18 years old, and persons outside the Kingdom of Saudi Arabia. A convenience sample of women with breast cancer was conducted through an electronic questionnaire. The researchers introduced the study aim, objectives, and the significance of the study to the participants, and the consent form was obtained from the participants at the beginning of the questionnaire as follows: Your answer to the questionnaire questions means that you agree to participate in this study. The questionnaire took approximately 5–7 min.

### 2.4. Tools for Data Collection

The data were collected using a descriptive cross-sectional electronic online questionnaire, which can reach a large number of participants. The participants can answer with complete privacy and anonymity, and it can cover every aspect of a topic. It is considered to be more reliable and offers a quick, practical, and cost-effective way to obtain the desired results and save data collectors’ time. On the other hand, electronic online questionnaires do not include open-ended questions. Therefore, participants cannot convey their feelings or emotions, some participant may not answer all the questions with credibility, and there is no chance for explanation or clarification of questions from the researchers when participants fill out the questionnaire. In this study, we used an adopted questionnaire from a previous study done in Korea to determine the level of awareness regarding BCRL [25].

The electronic online questionnaire consisted of a total of 23 questions in two languages; the participants could choose either Arabic or English. It consisted of four parts. The first part included demographic data (five items) such as age, body weight, height, region, and dominant hand. The second part concerned status of education about BCRL (three items), including the following: did they know about lymphedema, did they receive any explanation about lymphedema from health care providers before surgery, and did they receive any explanation about lymphedema from health care providers after surgery. The third part was the basic medical history of breast cancer (six items), including disease duration, lymphedema treatment, lymph node dissection, lymphedema diagnosis, if they received chemotherapy after surgery, and if they received radiotherapy after surgery. The fourth part covered BCRL level of awareness of risk factors and management (nine items), including upper limb hygiene, mild trauma to the upper limb, tight clothing or wearing bracelets, excessive use of upper extremities, weight gain, is lymphedema a disease that should be treated, is lymphedema completely curable, which activities help lymphedema occurrence, and which department can manage lymphedema. The participants’ responses were measured by answering multiple choice and yes or no questions.

### 2.5. Ethical Considerations

We obtained ethical approval from the Faculty of Nursing at King Abdul-Aziz University, Jeddah, Kingdom of Saudi Arabia. Ethical approval included the purpose of conducting this study, the questionnaire, and sampling method. We obtained the data from participants who were selected according to the following inclusion and exclusion criteria: adult women with breast cancer or women who are breast cancer survivors, their ages were 18 years and above, and they live in the Kingdom of Saudi Arabia. The exclusion criteria were males who have breast cancer, women with breast cancer under the age of 18 years old, and persons outside the Kingdom of Saudi Arabia.

An electronic online questionnaire was used that included informed consent, which was distributed through social media applications such as WhatsApp, Twitter, and Instagram.

### 2.6. Data Analysis

Descriptive analysis was performed after data collection using Statistical Package for Social Sciences (SPSS software) (IBM, Inc., Chicago, IL, USA) version 26 in the form of means, percentages, frequencies, and standard deviations.

## 3. Results

Of the study sample, 52.6% were between the ages of 18 and 35 years, 18.5% were between 36 and 45 years, 26.7% were between 46 and 65 years, and 2.2% were 65 years or above. Regarding weight, 47.4% of the participants weighed less than 60 kg, 47.4% weighed between 60 and 100 kg, and 3% weighed more than 100 kg. Regarding height, 71.1% of participants were less than 160 cm, 25.2% were between 160 and 170 cm, and 3.7% were more than 170 cm. Regarding region, 9.6% of participants lived in the Central Region, 71.9% of them lived in the Western Region, 6.7% of them lived in the Eastern Region, and 5.2% of them lived in the Southern Region. Of the study sample, 92.6% were right-hand dominant and 7.4% were left-hand dominant. (Table 1.)

Table 2 shows that only 29.6% of the study sample knew about lymphedema, 11.9 percent of them received a clarification by a medical team member prior to surgery regarding the likelihood of lymphedema, and 10.4 percent of them provided a description by a medical team member following surgery about the possibilities of lymphedema.

Table 3 shows that 51.1% of the study sample had lymphedema for less than 1 year, 16.3% of them had lymphedema for less than 2 years, 10.4% of them had lymphedema for less than 3 years, and 22.2% of them had lymphedema for more than 3 years. Of the study sample, 19.3% had received treatment for lymphedema, 9.6% had lymph node dissection surgery, 3.7% had been diagnosed with lymphedema, 8.1% had received chemotherapy after surgery, and 6.7% had received radiotherapy after surgery.

Table 4 shows that 62.2% of the study sample said that it is possible that poor hygiene of an upper limb can increase the risk of lymphedema occurrence, 67.4% of them said that it is possible that trauma of an upper limb can increase the risk of lymphedema occurrence, 54.8% of them said that it is possible that constriction of an upper limb by a bracelet or tight shirt can increase the risk of lymphedema occurrence, 36.3% of them said that it is possible that overuse of an upper limb could increase the risk of lymphedema occurrence, 70.4% of them said that it is possible that weight gain could increase the risk of lymphedema occurrence, 91.1% of them think that lymphedema is a disease that should be treated, and 11.1% of them said that lymphedema is a disease that cannot be completely cured. Further, 5.2% of the sample study believed that golf can help reduce the risk of lymphedema occurrence, and 9.6% of them said that tennis (using the affected upper limb) can help reduce the risk of lymphedema occurrence. Moreover, 45.2% of the study sample said that general surgery can manage lymphedema, 2.2% said that family medicine can manage lymphedema, 20.7% of them chose physical medicine and rehabilitation to manage lymphedema, and 1.5% said that orthopedic surgery can manage lymphedema.

## 4. Discussion

The study was done to investigate the level of lymphedema awareness among women with breast cancer in the Kingdom of Saudi Arabia, as it is considered to be a common complication among women with breast cancer. It was found that the majority of the participants in this study did not know about lymphedema (70.4%) (Table 2). A similar result was found in a study with a total of 108 participants who were women with breast cancer, indicating that the level of information provided to the participants was low [27]. Only 19.5% of the participants had declared that they were aware of lymphedema, and 80.5% of participants were neither informed nor educated about BCRL. So, the study suggests that the risk of developing lymphedema is increased in patients who do not receive information about lymphedema after surgery compared with those who had received such information [27]. This study presents a result similar to a previous review article, which showed that awareness of BCRL signs and symptoms for both patients and health care providers is needed to prevent the occurrence of lymphedema among women with breast cancer [23].

Furthermore, a study on a total of 103 women with breast cancer stated that knowledge of lymphedema was remarkably related to adherence of women with breast cancer to the recommended strategies of the treatment plan, and women who recognized lymphedema risk management showed greater adherence to the recommended strategies [22]. Likewise, a previous systematic review was done on four different studies in four different countries (Canada, United Kingdom, United States, and Japan). It found that the majority of participants with breast cancer had a significant lack of information regarding BCRL awareness, and the reason behind it is that the health care providers did not have the enough knowledge regarding BCRL. Therefore, the participants were uninformed about BCRL and the minority of participants who had information about BCRL obtained it from resources other than the health care providers [26]. In addition, a study was conducted on 116 women with breast cancer regarding their level of awareness of BCRL. The study found that the majority of the participants knew about lymphedema (69.82%), and 58.62% of the participants received an explanation about lymphedema from the medical team after surgery. However, 74.13% of the participants did not receive any explanation before surgery, similar to our study [25]. Moreover, a study on 250 women with breast cancer suggested that postoperative health programs on BCRL can help minimize morbidity [24]. Another study on 389 participants diagnosed with breast cancer found that the age of the participants was the major factor regarding the level of awareness of lymphedema symptoms and diagnosis. Participants between the ages of 50 and 59 years had the highest level of awareness regarding lymphedema symptoms (29.73%) and diagnosis (35.32%) [16]. In contrast to our study, an article including 145 women with breast cancer who had a mastectomy found that the majority of the participants (83%) had information on how to prevent lymphedema and that 79% received this information from a physiotherapist [28].

Analysis of the questionnaire used in this study shows that the most significant factor affecting participants’ level of awareness regarding BCRL is the lack of information about the possibility of BCRL occurrence, which was not provided by the medical team (before surgery: 88.1%; after surgery: 89.6%). A previous study suggested that an early educational program regarding BCRL awareness for women with breast cancer could be a major factor in preventing the development of lymphedema, and detecting the signs and symptoms of BCRL, early diagnosis, and proper treatment will lead to improving patients’ quality of life [16]. In addition, another significant finding that emerged from our study analysis is that 13 participants (9.6%) had lymph node dissection surgery, 11 (8.1%) had chemotherapy after surgery, 9 (6.7%) had radiotherapy after surgery, and 5 (3.7%) were diagnosed with lymphedema. This correlates with a previous study, which indicated that these factors are common causes for the development of BCRL in women with breast cancer [5].

This study revealed the emerging need for medical teams to provide women with breast cancer the necessary information regarding BCRL. Moreover, the findings of our study highlight the need for providing early education programs for women with breast cancer carried out by the nursing staff, who are considered to be important members of the health care team involved in the care plan and who are most in contact with the patients. The nursing staff must also have adequate knowledge regarding BCRL signs and symptoms, risk factors, and how to prevent it when caring for women with breast cancer in order to help and educate women with breast cancer to be more knowledgeable and to decrease the likelihood of developing BCRL.

## 5. Conclusions

This was the first study to investigate the level of awareness of BCRL among women with breast cancer in the Kingdom of Saudi Arabia. The study showed an extremely low level of awareness regarding BCRL among the women with breast cancer who participated in this study from different regions of the Kingdom of Saudi Arabia in comparison with another study in another country [25]. The most significant factor affecting the level of awareness of most participants is that no education was provided to the participants, and they did not receive any explanation about the possibility of BCRL occurrence before or after surgery from medical team members.

The limitations of this study include the small number of participants. Moreover, this study was conducted during the Coronavirus Disease 2019 (COVID-19) pandemic, which forced us to change the number of the target population and the method for data collection from face-to-face interviews (which would have been held in the hospital) to an electronic online questionnaire distributed through social media applications such as WhatsApp, Twitter, and Instagram. Therefore, the time required to conduct this study was limited, which was considered to be the most significant factor for us in our study. In addition to these limitations, there were limited numbers of recent studies about the level of awareness of BCRL globally. So, we were forced to use old studies as a reference in this research. Further, no studies had been conducted regarding the level of awareness of BCRL among women with breast cancer in the Kingdom of Saudi Arabia.

The study results indicate that there is an intense need for developing an early educational program regarding the possibility of BCRL occurrence for women with breast cancer, especially women who have undergone breast cancer surgery and have received chemotherapy or radiotherapy, as these are considered to be major risk factors for developing BCRL. This educational program should be provided during the early course of treatment by the medical team, as such information plays a significant role in preventing problems related to BCRL for women with breast cancer and will increase their awareness about signs, symptoms, and risk factors. Therefore, early detection and proper treatment should be provided to improve patients’ quality of life and to ensure a better outcome for women with breast cancer. We advise that medical teams should have a continuous educational program emphasizing especially the importance of the awareness of BCRL to educate and increase the knowledge of women with breast cancer and their caregivers.

In addition, as there is serious relationship between the level of awareness of BCRL and improving the quality of life of women with breast cancer, this study revealed that there is an emerging need for more studies to be conducted regarding the level of awareness of women with breast cancer about BCRL in the Kingdom of Saudi Arabia.

We recommend that future studies investigate the relationship between the level of lymphedema awareness and improving the quality of life of women with breast cancer and engage a greater number of participants.

## Figures and Tables

**Table 1:** Percentage and frequency allocation of the study sample by their sociodemographic characteristics (*n* = 135).

Characteristics	Category	Count	Total *n*%
Age	18–35 years	71	52.6%
	36–45 years	25	18.5%
	46–65 years	36	26.7%
	65 years and above	3	2.2%
Weight	Less than 60 kg	64	47.4%
	From 60 to 100 kg	64	47.4%
	More than 100 kg	4	3.0%
Height	Less than 160 cm	96	71.1%
	From 160 to 170 cm	34	25.2%
	More than 170 cm	5	3.7%
Region	Central Region	13	9.6%
	Western Region	97	71.9%
	Eastern Region	9	6.7%
	Southern Region	7	5.2%
	Northern Region	9	6.7%
Dominant hand	Right hand	125	92.6%
	Left hand	10	7.4%

**Table 2:** Frequency and percentage distribution of the study sample by status of education about breast-cancer-related lymphedema (BCRL) (*n* = 135).

Variable		Count	Total *n*%
1. Do you know about lymphedema?	Yes	40	29.6%
	No	95	70.4%
2. Did you receive an explanation about the possibility of lymphedema by a medical team member before surgery?	Yes	16	11.9%
	No	119	88.1%
3. Did you receive an explanation about the possibility of lymphedema by medical a team member after surgery?	Yes	14	10.4%
	No	121	89.6%

**Table 3:** Percentage and frequency of basic medical history of breast cancer (*n* = 135).

Variable	Category	Count	Total *n*%
1. Disease duration (time since breast cancer)	Less than 1 year	69	51.1%
	Less than 2 years	22	16.3%
	Less than 3 years	14	10.4%
	3 years and above	30	22.2%
2. Lymphedema treatment	Yes	26	19.3%
	No	109	80.7%
3. Did you have lymph node dissection surgery?	Yes	13	9.6%
	No	74	54.8%
4. Lymphedema diagnosis	Yes	5	3.7%
	No	87	64.4%
5. Did you receive chemotherapy after the surgery	Yes	11	8.1%
	No	62	45.9%
6. Did you receive radiotherapy after the surgery	Yes	9	6.7%
	No	63	46.7%

**Table 4:** Percentage and frequency for the study sample by their BCRL level of awareness of risk factors and management (*n* = 135).

Variable		Count	Total *n*%
1. Is it possible that poor hygiene of an upper limb can increase the risk of lymphedema occurrence?	Yes	84	62.2%
	No	51	37.8%
2. Is it possible that trauma of an upper limb can increase the risk of lymphedema occurrence?	Yes	91	67.4%
	No	44	32.6%
3. Is it possible that constriction of an upper limb by a bracelet or tight shirt can increase the risk of lymphedema occurrence?	Yes	74	54.8%
	No	61	45.2%
4. Is it possible that overuse of an upper limb can increase the risk of lymphedema occurrence?	Yes	49	36.3%
	No	86	63.7%
5. Is it possible that weight gain can increase the risk of lymphedema occurrence?	Yes	95	70.4%
	No	40	29.6%
6. Is lymphedema a disease that should be treated?	Yes	123	91.1%
	No	12	8.9%
7. Is lymphedema a disease that cannot be completely cured	Yes	15	11.1%
	No	120	88.9%
8. Which activity is helpful to reduce the risk of lymphedema occurrence (can choose more one answer)?	Golf	7	5.2%
	Tennis (using affected upper limb)	13	9.6%
	Swimming	0	0.0%
	Ping-pong (using affected upper limb)	0	0.0%
	Hard weight training	4	3.0%
	No idea	102	75.6%
	Not applicable	19	14.1%
9. Which department can manage lymphedema (can choose more one answer)?	General surgery	61	45.2%
	Family medicine	3	2.2%
	Physical medicine and rehabilitation	28	20.7%
	Orthopedic surgery	2	1.5%
	No idea	52	38.5%
	Other	5	3.7%
